# Development and application of a curcumin-cinnamon essential oil nanoemulsion agent against mycobacteria

**DOI:** 10.3389/fcimb.2025.1582416

**Published:** 2025-06-25

**Authors:** Zhihui Lei, Yixuan Ren, Jinyao Wang, Haisu Shi, Hu Lin, Hong Chen, Lijun Bi, Yutang Wang, Hongtai Zhang

**Affiliations:** ^1^ College of Food Science, Shenyang Agricultural University, Shenyang, Liaoning, China; ^2^ Institute of Food Science and Technology, Chinese Academy of Agricultural Sciences, Key Laboratory of Agro-products Processing, Ministry of Agriculture and Rural Affairs, Beijing, China; ^3^ Key Laboratory of RNA Biology, Institute of Biophysics, Chinese Academy of Sciences, Beijing, China; ^4^ Beijing Center for Disease Prevention and Control, Beijing, China; ^5^ College of Pulmonary and Critical Care Medicine, the 8th Medical Centre of Chinese PLA General Hospital, Beijing, China; ^6^ Guangzhou National Laboratory, Guangzhou, Guangdong, China

**Keywords:** curcumin, cinnamon essential oil, nanoemulsion, aerosols, mycobacteria, *Mycobacterium tuberculosis*

## Abstract

With the increasing prevalence of drug-resistant *Mycobacterium tuberculosis* (*M. tuberculosis*), the development of novel anti-mycobacterial agents has become urgent. In this study, a curcumin-cinnamon essential oil (Cur-CEO) nanoemulsion was developed with optimized preparation parameters, including a 10% CEO volume fraction, 7.5 minutes of ultrasonic treatment, and 350 W ultrasound power, yielding a particle size of 101.14 nm. The nanoemulsion demonstrated high encapsulation efficiency (90.2%) and stability, with a stability coefficient of 0.984. Structural analysis revealed a dense network structure of the nanoemulsion and amorphous forms of Cur and CEO, enhanced by hydrogen bonding and electrostatic interactions, which improved solubility and bioavailability. The Cur-CEO nanoemulsion exhibited potent antimicrobial activity against mycobacteria, demonstrating MIC values of 2 µg/mL and 0.25 µg/mL against *Mycobacterium smegmatis* and *M. tuberculosis*, respectively, representing a fourfold reduction compared to the CEO solution alone, owing to its ability to induce substantial damage to mycobacterial cell membranes and consequently enhance nucleic acid and protein leakage. Furthermore, the aerosol form of the nanoemulsion effectively inhibited both surface and airborne mycobacteria, with no significant changes in structural properties post-atomization. Lung deposition studies indicated that 75.6% of aerosol particles of the nanoemulsion reached the alveolar region, suggesting its potential as an inhalation agent. Additionally, the Cur-CEO nanoemulsion exhibited negligible effects on macrophage viability, maintaining a survival rate exceeding 85% even at concentrations up to 1250 ng/mL. These findings indicate that the Cur-CEO nanoemulsion, formulated using natural ingredients, holds significant promise as a food-grade antibacterial agent for the prevention and control of mycobacterial infections.

## 1 Introduction

Tuberculosis (TB) has emerged as a significant global public health challenge, further exacerbated by the growing issue of drug resistance in *M. tuberculosis*, underscoring the urgent need for a novel anti-mycobacteria agent. The essential oils (EOs), which are secondary metabolites of aromatic plants, have garnered attention for their anti-biofilm and antibacterial properties (Bajalan et al., 2017).

Cinnamon essential oil (CEO), a yellow, oily, non-toxic volatile substance extracted from cinnamon bark and leaves, contains cinnamaldehyde, which exhibits antibacterial (Rula and Talal, 2010), antiviral, and anti-mycobacterial activities (Sawicki et al., 2018). Beyond antibacterial effects, many EOs also possess antioxidant, anti-tumor, antifungal, and anti-inflammatory propertie (Iordana and Alina, 2021). However, their application in food and pharmaceuticals is limited by low water solubility, high volatility, pungent taste, and instability under light, temperature, and humidity.

Natural active products are widely used in medicine, food, cosmetics, and harmaceuticals (Harjo and C.Wibowo, 2004). Curcumin (Cur), a polyphenolic compound extracted from Curcuma longa L. rhizomes, is a rare diketone in the plant kingdom (Patton and Byron, 2007). Beyond its use as a seasoning and coloring agent, Cur exhibits various pharmacological effects, including antioxidant (Saadati et al., 2019), anti-inflammatory (Hewlings and Kalman, 2017), anticancer (Farhoudi et al., 2022), and antibacterial activities (Zheng et al., 2020). Studies suggest that Cur and its derivatives hold potential for TB treatment, either alone or in combination with other drugs (Baldwin et al., 2015). However, their traditional applications are constrained by low bioavailability and poor stability.

Nanotechnology, particularly nanoemulsion, offers a promising solution by enhancing stability, bioavailability, and targeting, thereby improving the antibacterial efficacy of natural substances (Hu and Luo, 2021).

Nanoemulsions, are employed to disperse EOs in aqueous systems (Targino de Souza Pedrosa et al., 2021). These systems, composed of water, oil, surfactants, and co-surfactants, exhibit thermodynamic stability and transparency (Xiaoqing et al., 2011). Nanoemulsions, with their small particle size and high stability, show great potential in drug delivery, particularly for lung-targeted therapies. Thus, developing a safe and effective nebulized drug delivery system putting Cur and CEO together via nanoemulsion technology is a viable approach for anti-mycobacteria.

This study aims to develop a stable Cur-CEO nanoemulsion system utilizing food-grade ingredients, analyze its structural characteristics, and evaluate its anti-mycobacterial activity and underlying mechanisms. Additionally, the research seeks to investigate the antibacterial efficacy of the nanoemulsion aerosol when applied to surfaces and dispersed in the air. Furthermore, the study will assess the deposition patterns of the nanoemulsion aerosol within the lungs and explore its potential as an innovative inhalation-based strategy for the prevention and control of mycobacterial infections.

## 2 Materials and methods

### 2.1 Preparation of the Cur-CEO nanoemulsion

The Cur-CEO nanoemulsion was prepared by dissolving 3 mg of curcumin (Cur) in 10% cinnamon essential oil (CEO) to form the oil phase. A mixture of 5% Tween 80, 5% medium-chain triglycerides (MCT), and 80% ultrapure water was ultrasonicated at 350 W for 7.5 minutes using an ultrasonic cell disruptor (JY92-IID; Shanghai Haozhuang Instrument Co., Ltd., China).

### 2.2 Determination of particle size

The droplet size distribution and PDI were measured using a Zetasizer Nano ZS (Malvern Instruments, UK) potential analyzer. The sample was pre diluted 100 times with PBS to avoid multiple scattering effects (Chen et al., 2021).

### 2.3 Measurement of stability and encapsulation efficiency

Determined by HPLC (Hypersil C18 column, 250 mm × 4.6 mm, 5 µm; mobile phase: acetonitrile-4% glacial acetic acid, 55:45; flow rate: 1.0 mL/min; detection: 425 nm) stability coefficient (K) and encapsulation efficiency (EE%). The stability was evaluated by centrifuging the nanoemulsion at 4 and 5000 xg for 30 minutes. Measure the curcumin content (W_1_) of the supernatant and calculate the stability coefficient (K) as K=W_1_/W_0_ (W_0_=total curcumin content). After centrifugation at 5180 xg for 20 minutes, the free curcumin (W_free_) was quantified, with EE (%)=(1-W_free_/W_0_)×100% (W_0_=total curcumin content).

### 2.4 Cryo-electron microscopy analysis

The nanoemulsion was rapidly frozen for 30 seconds, sublimated at −90°C for 10 minutes, and gold-sputtered for 60 seconds. It was observed using a low-temperature SEM (FEI Quanta 450; SU3500) at 5 kV and −140°C.

### 2.5 Analysis of membrane permeability

Mycobacterium cells (1 × 10³ CFU/mL) were treated with 0.5× MIC nanoemulsion for 96 hours. After centrifugation at 10,000 × g for 10 minutes, protein (Abs280) and nucleic acid (Abs260) content in the supernatant were measured (Wang et al., 2022).

### 2.6 Mycobacterium inhibitory activity

Bacterial suspension (1 × 10³ CFU/mL) was treated with nanoemulsion (128 µg/mL initial concentration) in a 96-well plate. MIC was the lowest concentration inhibiting visible growth. MBC was determined by plating clear wells on solid medium and incubating for 14 days (Sawicki et al., 2018).

### 2.7 Assay of Aerosol Inhibiting Mycobacteria

Surface Antibacterial Activity: Bacterial suspension on agar plates was exposed to atomized CEO and nanoemulsion in a sealed chamber. Bacterial concentration was compared pre-and post-treatment.

Airborne Antibacterial Activity: A TK-3 microbial aerosol generator dispersed bacteria into a chamber, and nanoemulsion aerosol was introduced. Bacterial growth was assessed post-incubation.

### 2.8 Aerodynamic Particle Size Distribution (APSD) Measurement

The APSD of the Cur-CEO nanoemulsion aerosol was measured using an FA-3 aerosol sampler (Jiangsu Changzhou Pusen Electronic Instrument Factory). The nanoemulsion was nebulized in a sealed chamber for 5 minutes, and the aerosol was collected on stages 1-7, washed with methanol. The mass median aerodynamic diameter (MMAD) and geometric standard deviation (GSD) were calculated using the Wu method (Doub et al., 2020).

### 2.9 Cytotoxicity assay

RAW 264.7 macrophages (5 × 10³ cells/mL) were treated with Cur-CEO nanoemulsion for 24 hours. CCK-8 reagent was added, and absorbance at 450 nm was measured. Cell viability (%) = (A test − A blank)/(A control − A blank) × 100%.

### 2.10 Statistical analysis

All experiments were performed in triplicate. Data were analyzed using one-way ANOVA, with Tukey’s test for significance (P < 0.05).

## 3 Results

### 3.1 Preparation and optimization of the Cur-CEO nanoemulsion

The Cur-CEO nanoemulsion was prepared by dissolving 3 mg of Cur in CEO to form the oil phase, followed by the addition of Tween 80 and MCT, which were thoroughly mixed. Ultrapure water was then incorporated, and the mixture was subjected to ultrasonic treatment to form the Cur-CEO nanoemulsion. Single-factor experiments were conducted to optimize the preparation conditions.

**Figure 1:**
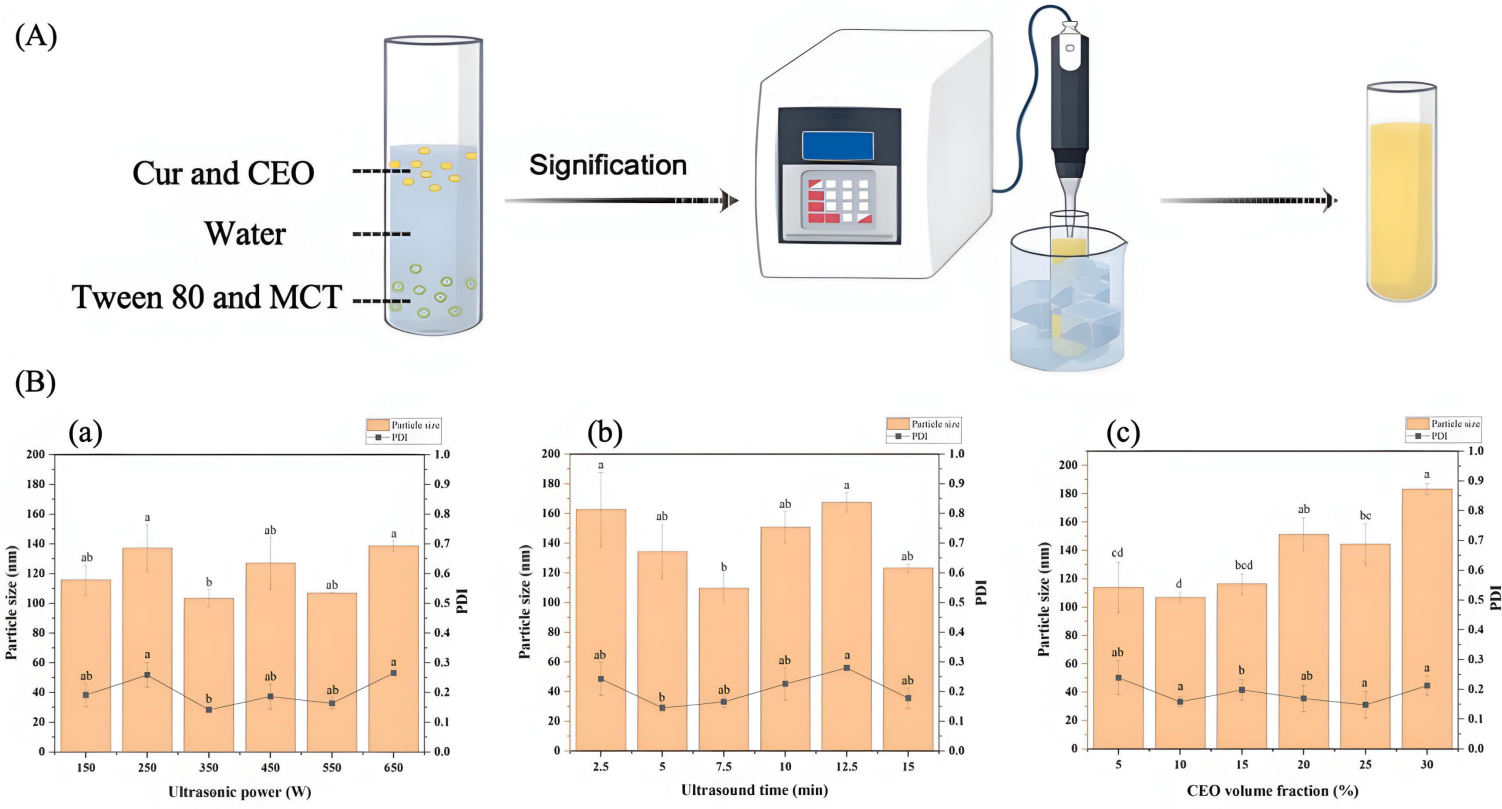
**(A)** Preparation process of curcumin-cinnamon essential oil (Cur-CEO) nanoemulsion; **(B)** Single-factor experiment results: (a) Effect of ultrasonication power, (b) Effect of ultrasonication time, and (c) Effect of cinnamon essential oil (CEO) volume fraction on particle size and PDI of the Cur-CEO nanoemulsion. Data shown are means ± SD (n = 3).

The effect of ultrasound power on particle size and polydispersity index (PDI) was evaluated, revealing that smaller particle sizes (103.3 nm and 106.2 nm) and lower PDI values (0.14 and 0.153) were achieved at 350 W and 550 W, respectively (**Figure 1Ba**). Considering the energy conservation and emulsion stability, 350 W was selected as the optimal ultrasound power. The influence of ultrasonic treatment time demonstrated that both particle size and PDI initially decreased with prolonged treatment but began to increase after a certain duration (**Figure 1Bb**). To avoid potential adverse effects from excessive exposure, the optimal ultrasonic treatment time was determined to be 7.5 minutes, yielding a particle size of 109.53 nm and a PDI of 0.165.

The volume fraction of CEO was also investigated, showing that increasing the CEO content led to a corresponding increase in particle size (**Figure 1Bc**). Due to the strong odor of CEO, a 10% volume fraction was selected as the optimal value, resulting in a particle size of 106.75 nm and a PDI of 0.158. Ultimately, the optimal preparation parameters for the Cur-CEO nanoemulsion were determined to be a CEO content of 10% (w/w), an ultrasonic treatment time of 7.5 minutes, and an ultrasound power of 350 W, yielding an average particle size of 101.14 nm.

Response surface methodology (RSM) was employed to further calculate the particle size of the nanoemulsion (**Supplementary Table 1**). The regression model equation for the particle size (Y) of the Cur-CEO nanoemulsion was derived as follows:


Y=133.36+18.67+36.27AB+31.4AC−7.8BC+32.18A2+29.53B2−28.77C2

where A represents ultrasound power, B represents ultrasound time, C represents the volume fraction of CEO, and Y represents particle size.

Analysis of variance (ANOVA) for the regression model indicated that the factors influencing particle size followed the order: CEO volume fraction (C) > ultrasound time (B) > ultrasound power (A) (**Supplementary Table 2**). Under the optimal preparation conditions, the predicted particle size of 98.76 nm closely matched the experimental result of 101.14 nm, confirming the reliability of the regression model for predicting the particle size of the Cur-CEO nanoemulsion.

### 3.2 Properties and structural characteristics of Cur-CEO nanoemulsion

The encapsulation efficiency (EE) and centrifugal stability of the Cur-CEO nanoemulsion were assessed by quantifying the Cur content. Initially, a standard curve for Cur was established using high- performance liquid chromatography (HPLC). The linear equations, y=0.0096x-0.0153, R^2^ = 0.9999 and y=142.93x-205.85, R^2^ = 0.9999, demonstrated a strong linear relationship for Cur concentrations ranging from 75-375 µg/mL and 1-50 µg/mL, respectively (**Supplementary Figure 1**).

The encapsulation efficiency of the nanoemulsion was evaluated by measuring the concentrations of Cur in the free phase (W_free_) and the total phase (W_total_), which were found to be 0.03 mg/mL and 0.307 mg/mL, respectively. The encapsulation efficiency was calculated to be 90.2%, indicating a highly efficient encapsulation capacity. This result confirms that the nanoemulsion effectively encapsulates the bioactive substance.

The centrifugal stability of the nanoemulsion was also investigated. Prior to centrifugation, the Cur content in the nanoemulsion was 0.307 mg/mL. After centrifugation at 5000 xg for 30 minutes, the nanoemulsion retained its clear and transparent appearance without phase separation, and the Cur content was measured at 0.302 mg/mL. The stability coefficient (K), calculated using the formula provided in the test method, was 0.984. This indicates that the Cur-CEO nanoemulsion is highly stable, with particles or oil droplets uniformly dispersed, and the system is resistant to phase separation or sedimentation.

**Figure 2:**
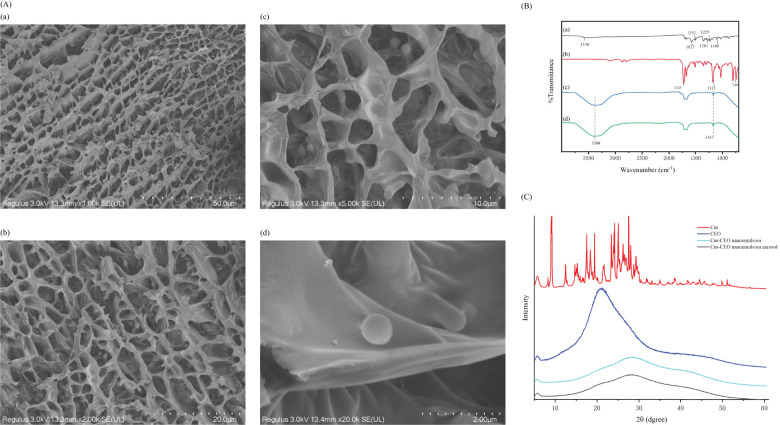
**(A)** Cur-CEO nanoemulsion Cryo-SEM. Observation of the internal structure of nanoemulsion using Cryo-SEM with shooting ratios of 50, 20, 10, and 2 µm for (a-d), respectively. **(B)** FTIR, Cur (a) CEO (b), Cur-CEO nanoemulsion (c), Cur-CEO nanoemulsion aerosol (d). **(C)** SAXS of curcumin (Cur), cinnamon essential oil (CEO), Cur-CEO nanoemulsion, Cur-CEO nanoemulsion aerosol.

Cur and CEO are highly hydrophobic polyphenols that tend to crystallize in aqueous solutions, limiting their solubility. The crystal structure of the Cur-CEO nanoemulsion was analyzed using small-angle X-ray scattering (SAXS). The SAXS diffraction pattern exhibited a broad peak between 10° and 40°, indicating that Cur and CEO exist in an amorphous form within the water phase, with minimal crystalline structure (**Figure 2C**). The diffraction peak of CEO shifted from approximately 15° to 20°, suggesting chemical interactions between Cur and the active components of CEO. These findings imply that the nanoemulsion preparation enhances the solubility of Cur and CEO, thereby improving their efficacy (Guo et al., 2021).

The internal structure of the Cur-CEO nanoemulsion was examined using cryo-electron microscopy (Cryo-SEM). The images revealed a dense network structure, which enhances the strength and stability of the nanoemulsion (**Figure 2A**). The droplets exhibited a regular spherical shape with a relatively uniform distribution and morphology, averaging approximately 100 nm in diameter. This result aligns with the particle size measurements obtained earlier.

The interaction between Cur and CEO in the nanoemulsion was investigated using Fourier transform infrared spectroscopy (FTIR). The results showed that the phenol peak of Cur shifted from 3570 cm^−1^ to 3380 cm^−1^, accompanied by a significant increase in intensity, indicating the formation of hydrogen bonds (**Figure 2B**) (Wang et al., 2024). The enhanced OH stretching band observed between 3400–3200 cm^−1^ is attributed to a synergistic effect of hydrogen bonding and electrostatic interactions (Paswan et al., 2024). These interactions not only contribute to the stability of the nanoemulsion but also facilitate the release of active ingredients, further enhancing bioavailability.

### 3.3 Inhibitory effect and mechanism of the Cur-CEO nanoemulsion on mycobacteria

The inhibitory effect of the Cur-CEO nanoemulsion on mycobacteria was evaluated through minimum inhibitory concentration (MIC) experiments. The MIC values of Cur solution, CEO solution, and Cur-CEO nanoemulsion were determined using 96-well microtiter plates. The results revealed that the Cur solution exhibited negligible inhibitory effects on *M. smegmatis* MC2–155 and *M. tuberculosis* H37Rv, with an MIC of 5000 µg/mL. The inclusion of rifampicin-a first-line anti-tuberculosis drug-as a positive control revealed a MIC of 0.125 µg/mL against *M. tuberculosis* H37Rv, which shows excellent consistency with previously reported data for this reference strain (Heinrichs et al., 2018). In contrast, both the CEO solution and the Cur-CEO nanoemulsion demonstrated significant inhibitory activity against mycobacteria. The MIC values for the CEO solution were 8 µg/mL and 1 µg/mL for *M. smegmatis* and *M. tuberculosis*, respectively. Notably, the MIC values for the Cur-CEO nanoemulsion were further reduced to 2 µg/mL and 0.25 µg/mL for *M. smegmatis* and *M. tuberculosis*, respectively (**Table 1**). These findings suggest that the synergistic interaction between Cur and CEO within the nanoemulsion enhances its inhibitory efficacy against mycobacteria compared to CEO alone. Additionally, the MBC/MIC ratio of the Cur-CEO nanoemulsion against mycobacteria was calculated to be 4, indicating its potent bactericidal activity (Sawicki et al., 2018).

**Table 1:** Minimum inhibitory concentration (MIC) of curcumin-cinnamon essential oil (Cur-CEO) nanoemulsion on *M. smegmatis* and *M. tuberculosis*.

Antibacterial agent	MIC (µg/mL)	
	*M. smegmatis*	*M. tuberculosis*
Cur-CEO nanoemulsion	2	0.25
CEO	8	1
Cur	5000	5000

Nucleic acids and proteins are critical macromolecules in bacterial cells, essential for sustaining cellular life (Dai et al., 2021). In this study, the CEO content in the CEO solution and the Cur-CEO nanoemulsion was standardized to the same concentration at 0.5 MIC for M. smegmatis. The results demonstrated that the Cur-CEO nanoemulsion induced significantly higher concentrations of nucleic acid and protein leakage (366.55 µg/µL and 8.0167 µg/µL, respectively) compared to the CEO solution (44.9 µg/µL and 4.47 µg/µL, respectively). These findings indicate that the Cur-CEO nanoemulsion causes substantial damage to the cell membrane of mycobacteria, further elucidating its enhanced antimicrobial activity.

### 3.4 Application of the Cur-CEO nanoemulsion aerosol against mycobacteria

The aerosol form represents a significant application method for the Cur-CEO nanoemulsion. The nanoemulsion was converted into aerosols using a mesh atomizer. The antibacterial efficacy of the Cur-CEO nanoemulsion aerosol was evaluated against surface mycobacteria. In a confined space of 40 cm³, 2 mL of the Cur-CEO nanoemulsion was atomized (flow rate ≥ 0.15 mL/min) onto a 9 cm plate containing 1 mL of *M. smegmatis* suspension (1 × 10³ CFU/mL). After 5 minutes of aerosol treatment, the plate was incubated for 5 days. No bacterial growth was observed on the plate. In contrast, a similar experiment using the CEO solution resulted in 875 CFU/mL of bacterial growth. These results demonstrate that the Cur-CEO nanoemulsion aerosol is significantly more effective in inhibiting mycobacteria on surfaces compared to the CEO solution alone.

The efficacy of the Cur-CEO nanoemulsion aerosol in inhibiting airborne mycobacteria was also investigated. 2 mL suspension of *M. smegmatis* (1 × 10³ CFU/mL) was sprayed into a 40 cm³ enclosed space using a TK-3 microbial aerosol generator. Subsequently, 2 mL of the Cur-CEO nanoemulsion was atomized (flow rate ≥ 0.15 mL/min) for 1 minute. Airborne *M. smegmatis* was collected using an aerosol particle size distribution collector, and each level of the collector plate (pre-coated with solid culture medium) was incubated for 5 days. No *M. smegmatis* colonies were observed on any of the plates, indicating that the Cur-CEO nanoemulsion aerosol effectively sterilizes mycobacteria in the air (**Table 2**).

**Table 2:** Cur-CEO nanoemulsion aerosol against *M.smegmatis* .

Level	Concentration of *M. smegmatis* suspension (CFU/mL)	
	Before treatment (control group)	After treatment (experimental group)
1	23	0
2	109	0
3	52	0
4	141	0
5	158	0
6	240	0
7	57	0
MOC	4	0

MOC: (The final microporous collector: level 8) stage is equipped with filter paper, so the effective interception particle size is 0.

Whether the aerosol after atomization has changed the properties and structural characteristics of nanoemulsion is also evaluated. The aerosols were collected and allowed to cool naturally into a liquid state. This liquid was subsequently analyzed using Fourier transform infrared spectroscopy (FTIR) and small-angle X-ray scattering (SAXS). The results revealed no significant changes, and the particle size (101.14 nm before atomization vs. 109.6 nm after) and Cur content (0.307 mg/mL before vs. 0.304 mg/mL after) remained nearly identical (**Figure 2B, C**). These findings confirm that the aerosol form retains the properties and structural integrity of the Cur-CEO nanoemulsion and highlight the potential of the Cur-CEO nanoemulsion aerosol as a powerful tool for controlling mycobacterial contamination on surfaces and in the air.

### 3.5 Deposition location and safety of Cur-CEO nanoemulsion aerosols in the lung

**Figure 3:**
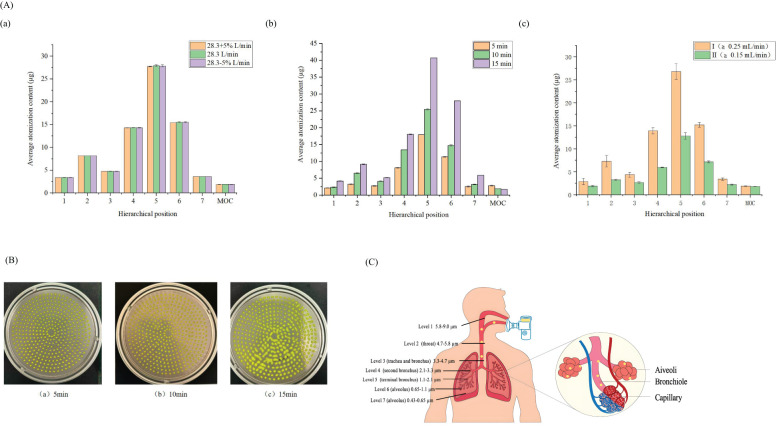
**(A)** Distribution of drug deposition at different levels, (a) Measurement of different flow velocities, (b) Different atomization times, (c) Atomizer at different speeds; P>0.05 **(B)** Collection situation of the fifth level at different atomization times; **(C)** Location of lung deposition at different levels; **(D)** Collection Results of Optimal Conditions; **(E)** Raw264.7 macrophage survival rate,* P<0.05.

To assess the potential of Cur-CEO nanoemulsion aerosols as inhalation agents, we evaluated their deposition patterns in the lung (Roberts and Mitchell, 2019). The FA-3 aerosol particle size distribution sampler was used to intercept aerosol particles at seven distinct size levels: 9, 5.8, 4.7, 2.1, 1.1, 0.65, and 0.43 µm. These levels correspond to specific deposition locations of the Cur-CEO nanoemulsion aerosols within the lung (**Figure 3C**). The amount of Cur collected at each level served as a marker for aerosol deposition.

First, we investigated the effects of flow rate, sampling duration, and aerosol grade on the particle size distribution. The results indicated that a flow rate of 28.3 L/min (± 5%) did not significantly affect the aerodynamic particle size analysis (**Figure 3A**). When comparing sampling durations of 5, 10, and 15 minutes, the 5-minute interval resulted in the least droplet adhesion at the sixth level of the collection tray (**Figure 3B**). Additionally, the fine particle fraction (FPF) of Grade II aerosols (≥0.15 mL/min) was higher than that of Grade I (≥0.25 mL/min), suggesting that aerosols at 0.15 mL/min are more effectively delivered to the lungs (**Table 3**).

**Table 3:** Results measured at different flow rates, times, and atomizer rates.

Variable	ISM (µg)	FPD (µg)	FPF (%)	MMAD (µm)	GSD	P
28.3-5% L/min	79.34	62.22	78.42	1.82	2.08	–
28.3 L/min	79.53	62.37	78.42	1.82	2.08	>0.05
28.3 + 5% L/min	80.07	62.88	78.52	1.82	2.08	>0.05
5 min	50.80	40.06	78.86	1.57	2.20	–
10 min	79.96	57.96	80.54	1.74	2.08	>0.05
15 min	112.85	91.89	81.43	1.74	2.04	>0.05
I≧0.25 mL/min	79.92	62.80	78.58	1.82	2.08	–
II≧0.15 mL/min	58.66	47.02	80.10	1.70	2.11	>0.05

The Cur-CEO nanoemulsion aerosol was prepared at an atomizer flow rate of ≥0.15 mL/min and collected for 5 minutes using the FA-3 sampler at a flow rate of 28.3 L/min. The hierarchical content of curcumin at each level was then determined. The results revealed that 75.6% of the particles in levels 4-7 (3.3-0.43 µm) successfully reached the human alveoli, indicating that the majority of the nanoemulsion was deposited in the alveolar region (**Table 4**). According to Baoshun et al (Baoshun and Chantal, 2011), 15.5% of the Cur-CEO nanoemulsion aerosols had an aerodynamic diameter of approximately 3 µm (fourth stage), suggesting that a significant portion of the aerosol was deposited in the alveolar sac during the first respiratory cycle. Furthermore, 38% of the total sediment at level 5 consisted of particles that could be engulfed by macrophages (**Table 4**) (Patton and Byron, 2007). Once internalized by macrophages, the Cur-CEO nanoemulsion could be transported more precisely to areas infected by *Mycobacteria*, thereby enhancing lung concentration and therapeutic efficacy.

**Table 4:** Collection Results of Optimal Conditions .

Level	1 (µg)	2 (µg)	3 (µg)	Mean value (µg)	Cumulative quantity (%)	RSD (%)
1	1.98	1.99	1.96	1.98	5.19	0.77
2	3.36	3.38	3.37	3.37	8.83	0.30
3	2.54	2.54	2.55	2.54	6.67	0.23
4	5.92	5.92	5.91	5.92	15.53	0.10
5	13.28	13.31	13.30	13.30	34.88	0.11
6	7.15	7.03	7.06	7.08	18.57	0.88
7	2.12	2.13	2.13	2.13	5.59	0.27
MOC	1.81	1.82	1.83	1.82	4.78	0.55
ISM	38.16	38.12	38.11	38.13	100	0.07

ISM, total collection of CI devices (area under APSD curve); MOC, (The final microporous collector: level 8) stage is equipped with filter paper, so the effective interception particle size is 0.

To evaluate the safety of the Cur-CEO nanoemulsion, we assessed its cytotoxicity on RAW 264.7 macrophages, which play a critical role in *Mycobacteria* infection in the lung (Chen et al., 2019). Cell viability was measured using the CCK-8 assay after 24 hours of exposure to varying concentrations of the nanoemulsion. The results demonstrated that the Cur-CEO nanoemulsion had minimal impact on macrophage viability, with survival rates exceeding 85% even at concentrations up to 1250 ng/mL (P<0.05) (**Figure 3D**). However, when the concentration was increased to 2500 ng/mL, the survival rate of RAW 264.7 macrophages decreased to 68.71%.

These findings suggest that the Cur-CEO nanoemulsion is a promising inhalation agent with favorable lung deposition characteristics and low cytotoxicity at therapeutic concentrations.

ISM: total collection of CI devices (area under APSD curve); FPD: mass less than 5 µm (successfully deposited lung contents); FPF: FPD as a percentage of the total particle collection (control sedimentation content); MMAD: particle size when APSD cumulative mass reaches 50% (mass median diameter); GSD: geometric standard deviation (discreteness of APSD curve). The 28.3-5% L/min, 5 min, and I ≥0.25 mL/min were used as the control values for flow rate, time, and atomizer flow rate, respectively.

## 4 Discussion

This study successfully developed a Cur-CEO nanoemulsion with a particle size of 101.14 nm and a stability coefficient of 0.984, demonstrating potent antimicrobial activity against mycobacteria. The minimum inhibitory concentration (MIC) values were 2 µg/mL for *M. smegmatis* and 0.25 µg/mL for *M. tuberculosis*. Furthermore, the aerosol formulation of the nanoemulsion not only effectively inhibited both surface and airborne mycobacteria but also showed promising potential as an inhalation agent, with 75.6% of aerosol particles reaching the alveolar region. Cytotoxicity assays confirmed its safety profile at therapeutic concentrations.

The findings highlight that the optimized preparation parameters and structural stability of the nanoemulsion ensure its practical applicability. We identified that high concentrations of the CEO phase content may hinder the formation of an effective emulsifier layer, leading to droplet aggregation, with the maximum particle size (183.23 nm) and polydispersity index (PDI) (0.212) observed at a 30% CEO volume fraction. The formation of a dense network structure in the CEO nanoemulsion is attributed to increased hydrophobicity from the Cur-CEO combination and the exposure of surface hydrophobic groups during ultrasonication (Dianhui et al., 2019). Excessive ultrasonication power can damage and reaggregate nanoemulsion droplet structures, increasing particle size, while prolonged ultrasonication intensifies droplet collisions, leading to particle aggregation (Wang et al., 2022).

Curcumin (Cur), a hydrophobic polyphenol, tends to crystallize in aqueous solutions, limiting its solubility. The diffraction peak of Cur-CEO shifted from 15° to 20°, indicating interactions between Cur and CEO. The stable nanoemulsion prevented Cur precipitation, with amorphous structures enhancing Cur solubility and efficacy.

The results also revealed a synergistic interaction between Cur and CEO within the nanoemulsion, significantly enhancing antimicrobial efficacy compared to CEO alone. While Sawicki et al (Sawicki et al., 2018). reported an MIC value of 8 µg/mL for CEO against M. tuberculosis, our Cur-CEO nanoemulsion exhibited significantly lower MIC values (2 µg/mL and 0.25 µg/mL) despite containing only 10% CEO. This enhanced efficacy likely results from the Cur-CEO synergy and the dense network structure of the nanoemulsion, potentially through increased microbial membrane permeability or combined targeting of metabolic pathways.

The aerosol form of the Cur-CEO nanoemulsion demonstrated superior efficiency in inhibiting mycobacteria compared to the nanoemulsion state, underscoring its potential for environmental and clinical applications. Evaluation of lung deposition revealed that the aerosol particle size and the unchanged structure and properties of the Cur-CEO nanoemulsion enabled effective drug delivery to the alveolar region, a critical site for mycobacterial infections.

This study has several limitations. The long-term stability of the nanoemulsion under various conditions was not assessed,and cytotoxicity tests were conducted on a limited number of samples without evaluations at the immune system or organ levels. While the membrane disruption hypothesis provides a plausible mechanism for the observed antimicrobial activity, the deeper mechanistic insights-such as membrane potential dynamics or ultrastructural characterization of treated mycobacteria-remain unexplored. *In vitro* lung deposition studies did not assess absorption efficiency, release rate, or residence time in animal models.

Future studies should prioritize addressing these limitations while advancing the translational potential of the Cur-CEO nanoemulsion. Investigations into long-term stability under diverse storage conditions and *in vivo* validation of lung deposition and antimicrobial efficacy are essential. The development of advanced imaging modalities, such as cryo-electron microscopy with BSL-3-compatible protocols, could elucidate membrane interaction mechanisms that remain unresolved. Expanding the evaluation to other mycobacterial species and exploring combination therapies with existing antimicrobial agents would further strengthen its clinical relevance. Further optimization of the aerosol delivery system to enhance lung targeting and minimize cytotoxicity at higher concentrations is also recommended.

While synthetic nanoformulations such as Huang et al.’s antibiotic-loaded outer membrane vesicles (OMVs) have demonstrated efficacy against Gram-negative pathogens (Zheng et al., 2020), our Cur-CEO nanoemulsion represents a paradigm shift in antimicrobial strategy by leveraging natural food-grade components with multi-target synergy-a design that minimizes resistance risks and enhances biosafety for pulmonary delivery. The potent antimicrobial activity and structural stability of the Cur-CEO system position it as a promising candidate for auxiliary anti-tuberculosis treatment, particularly through its aerosolized form, which enables targeted lung delivery with 75.6% alveolar deposition efficiency. Further optimization of the aerosol system to refine lung targeting and mitigate cytotoxicity at elevated doses will be essential for clinical translation. Beyond therapeutic applications, the formulation’s stability and environmental compatibility also support its potential use in controlling mycobacterial contamination in healthcare settings, bridging the gap between natural product therapeutics and advanced nanomedicine.

## Data Availability

The original contributions presented in the study are publicly available. This data can be found here:10.6084/m9.figshare.29365091.
