# Supplementary figures and images for: Development and application of a curcumin-cinnamon essential oil nanoemulsion agent against mycobacteria
Source: Front Cell Infect Microbiol. 2025 Jun 25;15:1582416. doi: 10.3389/fcimb.2025.1582416 (PMC12238011; doi:10.3389/fcimb.2025.1582416)

Supplementary Figure 1

(a)

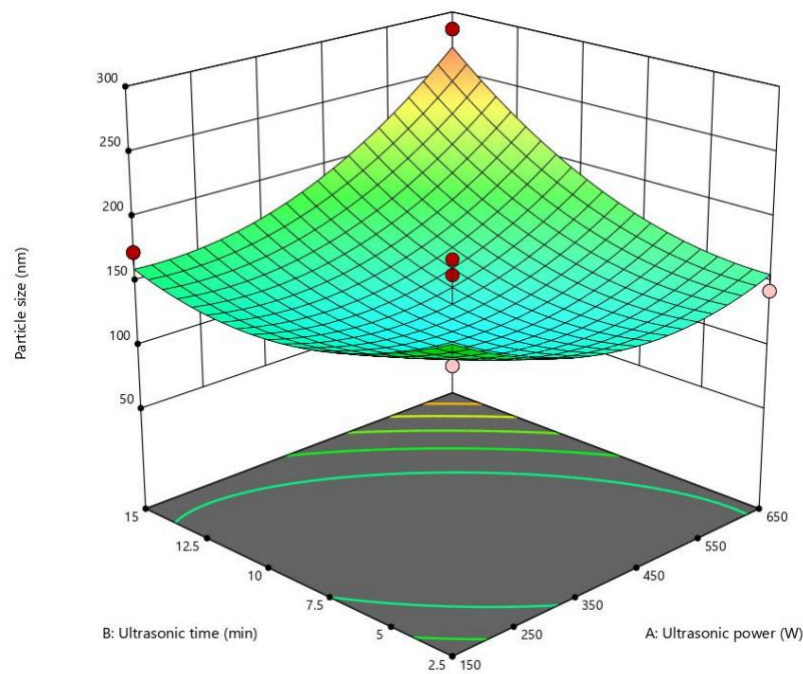

(b)

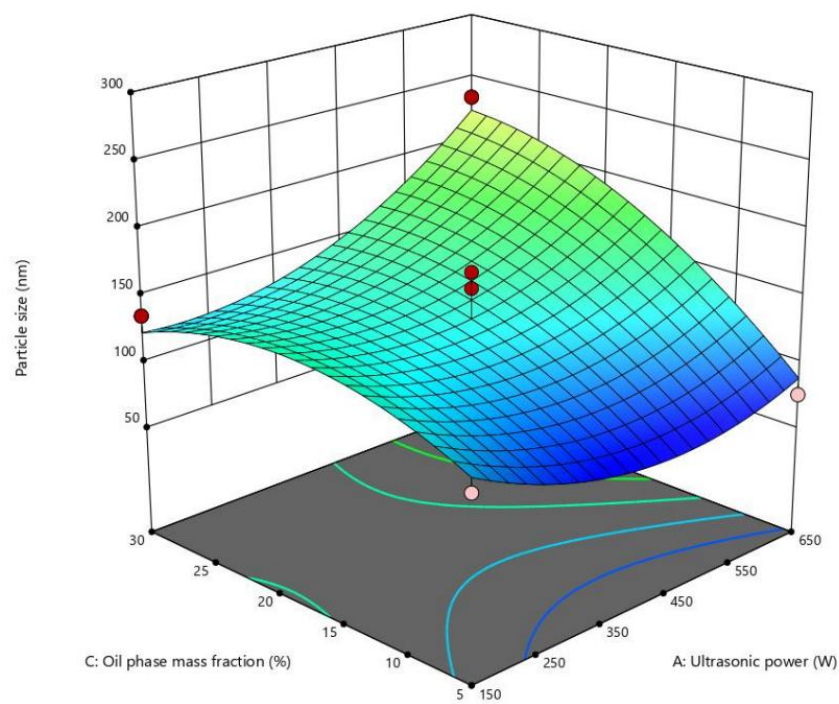

(c)

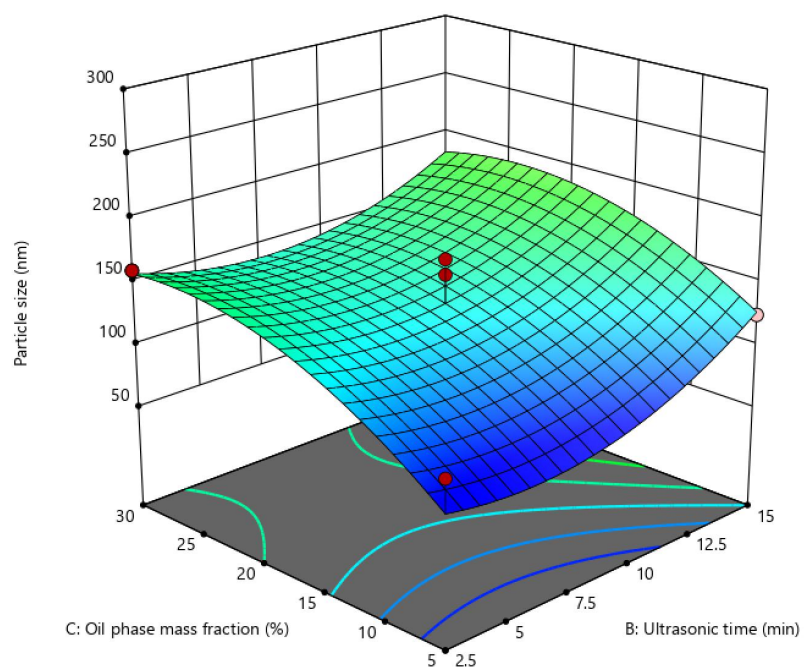

Supplement: Supplementary file 1 [file Image1.pdf]
