# Supplementary material for: Development and application of a curcumin-cinnamon essential oil nanoemulsion agent against mycobacteria
Source: Front Cell Infect Microbiol. 2025 Jun 25;15:1582416. doi: 10.3389/fcimb.2025.1582416 (PMC12238011; doi:10.3389/fcimb.2025.1582416)
Supplement: Supplementary file 2 [file Table1.pdf]

Supplementary Table 1 Response-surface design and results

Std	Ultrasonic power (W)	Ultrasound time (min)	Volume fraction of CEO (%)	Particle size (nm)
1	650	2.5	17.5	176.9
2	150	2.5	17.5	143.7
3	650	15	17.5	173.9
4	150	15	17.5	285.8
5	650	8.75	5	102.65
6	150	8.75	5	75.195
7	650	8.75	30	135.55
8	150	8.75	30	233.7
9	400	2.5	5	95.135
10	400	15	5	124.25
11	400	2.5	30	159.6
12	400	15	30	157.5
13	400	8.75	17.5	156.3
14	400	8.75	17.5	168.6
15	400	8.75	17.5	104.8
16	400	8.75	17.5	104.6
17	400	8.75	17.5	132.5
