# Supplementary material for: Development and application of a curcumin-cinnamon essential oil nanoemulsion agent against mycobacteria
Source: Front Cell Infect Microbiol. 2025 Jun 25;15:1582416. doi: 10.3389/fcimb.2025.1582416 (PMC12238011; doi:10.3389/fcimb.2025.1582416)
Supplement: Supplementary file 3 [file Table2.pdf]

Supplementary Table 2 Analysis of variance for the regression model

Source of variance	Sum of squares	Freedom	Variance	F value	P value	Significance
Model	37336.17	9	4148.46	4.77	0.0257	*
A	2789.86	1	2789.86	3.21	0.1164	
B	3449.07	1	3449.07	3.97	0.0867	
C	10448.44	1	10448.44	12.01	0.0105	**
AB	5263.50	1	5263.50	6.05	0.0435	*
AC	3944.15	1	3944.15	4.54	0.0707	
BC	243.52	1	243.52	0.28	0.6130	
A^2	4361.07	1	4361.07	5.01	0.0601	
B^2	3672.13	1	3672.13	4.22	0.0790	
C^2	3484.96	1	3484.96	4.01	0.0854	
Residual items	6087.53	7	869.65			
Omission item	2675.88	3	891.96	1.05	0.4636	
Pure error	3411.65	4	852.91			
Total	43423.70	16				

* indicates significant difference at $P < 0.05$; ** indicates highly significant difference at $P < 0.01$.
